# Switching Homes: How Cancer Moves to Bone

**DOI:** 10.3390/ijms21114124

**Published:** 2020-06-09

**Authors:** Marco Ponzetti, Nadia Rucci

**Affiliations:** Department of Biotechnological and Applied Clinical Sciences, University of L’Aquila, 67100 L’Aquila, Italy; marcopon@univaq.it

**Keywords:** bone metastasis, extracellular vesicles, exosomes, tumour dormancy, vicious cycle, premetastatic niche, hypoxia

## Abstract

Bone metastases (BM) are a very common complication of the most prevalent human cancers. BM are extremely painful and may be life-threatening when associated with hypercalcaemia. BM can lead to kidney failure and cardiac arrhythmias and arrest, but why and how do cancer cells decide to “switch homes” and move to bone? In this review, we will present what answers science has provided so far, with focus on the molecular mechanisms and cellular aspects of well-established findings, such as the concept of “vicious cycle” and “osteolytic” vs. “osteosclerotic” bone metastases; as well as on novel concepts, such as cellular dormancy and extracellular vesicles. At the molecular level, we will focus on hypoxia-associated factors and angiogenesis, the Wnt pathway, parathyroid hormone-related peptide (PTHrP) and chemokines. At the supramolecular/cellular level, we will discuss tumour dormancy, id est the mechanisms through which a small contingent of tumour cells coming from the primary site may be kept dormant in the endosteal niche for many years. Finally, we will present a potential role for the multimolecular mediators known as extracellular vesicles in determining bone-tropism and establishing a premetastatic niche by influencing the bone microenvironment.

## 1. Introduction

Metastatic bone disease is currently considered incurable [1]. Bone metastases (BM) are extremely common, and patients report unbearable pain in late stages, which makes palliative therapy with analgesics of the highest grade of the World Health Organisation analgesic ladder, i.e., strong opioids, a necessity [2]. BM incidence has been reported to be 65–90% in prostate cancer, 65–75% in breast cancer, 17–64% in lung cancer and 10% in colorectal cancer [3,4]. In addition to pain, common complications include pathological fractures, spinal cord and nerve compression syndromes and hypercalcaemia, which can lead to kidney dysfunction, cardiac arrhythmias and death [5]. However, it is important to notice that at least in breast cancer patients, BM generally present with better prognosis compared to visceral metastases. Therefore, improving the quality of life and reducing skeletal-related events (SREs) of BM patients would allow them to live close-to-normal lives, both in span and quality.

There are three main ways through which cancer cells manifest their presence in the bone microenvironment: osteosclerotic (a.k.a. osteoblastic) lesions, osteolytic lesions and mixed lesions [6]. Osteosclerotic lesions are typically related to prostate cancer BM and are identified as radio-dense spots on x-ray, while osteolytic lesions are present in BM from breast and most other types of cancers and are visually apparent as voids in areas where bone should be present [5,6]. Nowadays, the general idea is that even osteosclerotic lesions have an osteolytic component, that is either local, which gives rise to mixed lesions or can even be located far from the osteosclerosis. In fact, the consensus is that a primary osteolytic lesion is required to give rise to the osteosclerotic one, which explains why antiresorptive drugs such as bisphosphonates work also against this type of metastasis [7,8]. Intriguingly, breast cancer lesions can also present with mixed features [7,8].

The purpose of this review is to give an overview of the field, presenting well-established concepts, as well as new research that might be important for its future.

## 2. Cellular Players in the Bone/Bone Marrow Tumour Microenvironment

Osteoblasts are the cells that produce and mineralise bone matrix [9]. They arise from the differentiation of mesenchymal-lineage progenitors after activation of key osteoblast differentiation genes, with the master gene being Runt-related transcription factor 2 (Runx2) [2,10,11]. Runx2 can activate transcription of osteoblast-characterising genes, such as bone-specific alkaline phosphatase (BALP), osteocalcin (OCN), Collagen I and bone sialoprotein (BSP). Commitment towards the osteogenic program is driven by members of the Wingless-type MMTV integration site (Wnt) family, which activates the β-catenin pathway in osteo/chondroprogenitors in a paracrine fashion, leading to the aforementioned activation of Runx2 [12,13,14]. Osteoblasts are the major paracrine regulators of osteoclasts formation and function by producing a plethora of growth factors, cytokines and chemokines, which however can be exploited by cancer cells for their growth and engraftment, as we will discuss in detail below.

After osteoblasts finish their life cycle, they have three possibilities: (i) undergoing apoptosis, (ii) becoming a quiescent bone-lining cell or (iii) differentiating into osteocytes [9,15]. When the program to follow the latter option is activated, osteoblasts depose bone matrix around themselves, until the point where they are encased in it, becoming bona fide osteocytes [15,16,17]. This cell type vastly outnumbers the other bone-resident ones, and its main function is sensing mechanical variations in the microenvironment through a complex network of interconnected canaliculi, able to perceive shear stress by means of the primary cilium, which requires a protein complex composed by the cilium and the cilia-associated proteins Polycystin 1 and 2 [18,19,20]. Thanks to their sensing features, osteocytes are able to coordinate (and in specific circumstances, perform) bone resorption or deposition in response to both physiological environmental cues such as mechanical loading or lactation, as well as pathological ones, including micro- and macroscopic fractures [18,21,22,23,24]. Molecularly, osteocytes do this mainly through sclerostin (SOST), an antagonist of the canonical Wnt pathway that reduces bone formation when it is not needed [15] and RANKL, which allows them to control osteoclastogenesis as well [25].

Osteoclasts are multinucleated, giant, bone-resorbing cells [26]. At variance with osteoblasts, they derive from haematopoietic precursors of the monocyte/macrophage lineage, and their differentiation is a multistep process involving the activation of a series of transcription factors, including Microphthalmia Transcription Factor (MITF) and PU.1 [27,28,29]. In the last stages, osteoclast precursors express the macrophage-colony stimulating factor (M-CSF), which binds to its receptor c-fms eventually promoting their survival and growth. Costimulation by receptor activator of nuclear factor kappa B (NfĸB) ligand (RANKL) leads to activation of NfĸB, which in turns stimulates nuclear factor of activated T-cells, cytoplasmic, calcineurin-dependent (NFATc1), leading to fusion of precursors and generation of mature, resorbing osteoclasts [30,31,32,33]. Moreover, a plethora of inflammatory cytokines regulates osteoclastogenesis [34]. This feature is often exploited by cancer cells to induce osteoclastic activity and bone resorption [35].

A resident population of bone and bone marrow macrophages (BMMs) is present in both these tissues [34]. The formers are also known as osteal macrophages or osteomacs. Both cell types are F4/80-positive and TRAcP-negative in mice and are able to form functional osteoclasts in vitro [36]. Intriguingly, osteomacs have also been shown to produce BMPs, thus controlling osteoblast activity in vitro and in vivo [37,38]. They represent almost 20% of the bone marrow cells, and together with BMMs can become tumour-associated macrophages, eventually supporting tumour growth and immune escape, which is particularly important in the bone marrow, as it has been suggested in prostate cancer [39]. However, the role of these cells in breast cancer bone metastasis and dormancy is still to be elucidated.

Endothelial cells in the bone marrow have peculiar features, being organised in fenestrated sinusoids that allow for relatively easy intra- and extravasation of bone marrow and circulating cells, respectively [40]. The cross-regulation between bone cells and endothelial cells is known, as well as the concept that angiogenesis and osteogenesis are coupled during development and bone regeneration, where bone cells produce angiotropic factors, and endothelial cells produce angiocrine factors that stimulate bone modelling [41,42]. Importantly, bone marrow endothelial cells have been demonstrated to be involved in keeping cancer cells and HSCs dormant [40,43]. Recently, bone marrow endothelial cells have been suggested to promote prostate cancer progression through the CCL5/CCR5/autophagy axis, which is even able to reduce androgen receptor expression [44]. Furthermore, a recent emerging concept proposed by Lin and colleagues [45], is that endothelial cells can transdifferentiate into osteoblast-like cells in the metastatic prostate cancer context, thus inducing or contributing to osteosclerotic metastases [45].

Even though the commonly used mouse models have almost exclusively red marrow in their long bones, in adult humans this is mostly replaced by adipose yellow marrow. It is, therefore, conceivable that being the most abundant cell type in adult bone marrow, bone marrow adipocytes (BMAs) could have a crucial role in metastasis. In normal bone physiology, BMAs and osteoblasts both come from a common progenitor, i.e., the bone marrow mesenchymal stromal/stem cell [46], and a differentiation shift from osteoblasts to adipocytes is thought to be one of the determining factors in the bone mass accrual reduction during ageing [47]. Moreover, BMA-derived fatty acids are able to increase osteoclast differentiation and survival [48,49]. BMAs also support haematopoiesis, driving differentiation of CD34^+^ precursors towards the myeloid and lymphoid lineages [50]. In cancer, numerous reports suggest that BMA-derived fatty acids and factors such as fatty acids binding protein 4 (FABP4) and leptin, might drive tumour progression in prostate and breast cancer [51,52,53,54]. Intriguingly, adipose tissue-derived leptin has been positively associated with PI3K/Akt pathway activation and radio/chemotherapy resistance in cancer cells, further confirming the importance of BMAs in the bone marrow metastatic microenvironment [55,56]. In addition to this, as stated, BMAs can increase osteoclast activity and decrease osteoblast function, while providing nutrients for cancer cells in the form of fatty acids. This has been shown to be happening in metastatic cancer, through at least the PTH1R/PTHrP signalling pathway [47,53]. Although BMAs are also thought to be part of the endosteal niche, limited or no evidence is present to date about their role in cancer or HSC dormancy/reactivation [57].

## 3. Bone Metastasis: Established and Novel Concepts

Stephen Paget, more than 130 years ago, gathered data from over 700 autopsies of patients who died of cancer, and noticed a discrepancy between the metastatisation probability between similarly blood-perfused organs, such as spleen vs. liver. For breast cancer patients he stated that “In cancer of the breast the bones suffer in a special way, which cannot be explained by any theory of embolism alone” [58]. He stated that this was probably because the “seed” (i.e., the cancer cell) found a more favourable “soil” (i.e., the microenvironment) in certain organs, and, therefore, metastasis was more frequent in those districts [58]. This was a strong paradigm shift, since although Paget himself stated that the “seed” is more important, studying the “soil” could give valuable insights. Today, we know bone is a particularly attractive milieu for cancer cells: the bone matrix contains an abundance of growth factors of the transforming growth factor (TGF)-β superfamily, insulin-like growth factors (IGFs), bone morphogenetic proteins (BMPs), platelet-derived growth factor (PDGF), fibroblast growth factors (FGFs) and calcium (Ca^2+^) [59]. Importantly, these factors are not only stored in the bone matrix, but are also actively produced by osteoblasts [2,60].

### 3.1. “Virtuous” vs. “Vicious” Cycle of the Bone

Bone tissue undergoes continuous cycles of matrix renewal, which is carried out by bone-resident cells in a process termed “bone remodelling” [61]. To accomplish this process properly, bone cells cross-communicate in a tightly controlled manner, which guarantees the well-keeping of the delicate balance between bone deposition and resorption [2,15,62,63]. The cycle starts with a stimulus (mechanical or molecular) that primes periosteal or endosteal bone-lining cells, which move away from each other, exposing the bone surface to be remodelled, to the action of osteoclasts. This stimulates osteoclast precursors, which differentiate into osteoclasts in situ, attach and seal the exposed bone portion, and degrade it [26,64]. The consequence is the release of growth factors and chemoattractants, that summon reverse cells, macrophage-like cells that clean out the resorbed lacuna of debris and promote osteoblast differentiation. The latter phase is the longest one and culminates in the newly formed bone being covered in bone-lining cells again, closing the so-called “virtuous cycle” of bone [26,61]. Being such a well-structured process, which involves a large number of molecular players, cancer cells can hijack it in different phases to promote their own growth. Whatever the type of modification to this cycle, when a cancer cell corrupts it to its own benefit, the “virtuous cycle” becomes “vicious”, thus marking the establishment of bone metastasis [5,6].

In the “osteoblastic vicious cycle”, prostate cancer cells migrate to bone, and persuade osteoblasts to produce large amounts of growth factors-rich osteoid, which is then mineralised into primary bone, that never becomes lamellar [65]. Prostate cancer cells do this through several secreted and membrane factors, including Insulin-like growth factor (IGF), WNT1 and 3A, tumour necrosis factor (TNF)-α, BMPs, Endothelin (ET)-1 and parathyroid hormone-related peptide (PTHrP) [3,65,66,67,68,69]. In turn, osteoblasts produce factors that favour tumour growth, such as IGFs, chemokines, interleukin (IL)-6 and TGF-β [70]. Moreover, osteoblasts also promote osteoclast differentiation and activity through production of RANKL and M-CSF, which further stimulates the release of IGF-1, BMPs, PDGF, TGF-β from the bone matrix because of osteoclastic bone resorption, hence promoting tumour growth, restarting the vicious cycle [2,4,6,60,65,66]. Additionally, Ca^2+^ ions released from the bone matrix by osteoclasts have a role in the vicious cycle, since, in addition to causing potentially fatal hypercalcaemia, they can stimulate calcium-sensing receptor (CaSr)-positive cancer cells, which promotes their growth, apoptosis escape ability, and PTHrP production [71,72].

In the “osteolytic vicious cycle”, tumour cells produce factors (i.e., IL-6, TNF-α, IL-1β) that stimulate differentiation of osteoclast precursors into mature osteoclasts directly, or indirectly, such as PTHrP, that acts by increasing osteoblastic RANKL production, eventually stimulating osteoclast formation [73]. the final outcome is an exacerbation in bone resorption, which is not balanced by bone deposition [7,60,65], causing the release of the aforementioned growth factors encased in the bone matrix, which stimulate tumour growth, closing the vicious cycle.

### 3.2. From Primary Site to Bone: A Bumpy Ride

To establish the vicious cycle, cancer cells first have to migrate from the primary site to the bone, which is classically simplified in the following steps: (i) local invasion, (ii) transfer to bloodstream or lymphatic system which requires the ability to intravasate and survive in it, (iii) arrest and extravasation in distant site, (iv) immune resistance and establishment of a supportive niche in the secondary site (settlement), (v) activation of growth and overt metastasis, which unfortunately happens in a short period of time in the majority of cases, but can also have a latency of years [74]. All these steps make metastatisation a difficult journey, thus explaining the very low efficiency of the process, which is estimated to be less than 1%, with authors reporting values as low as 0.01% metastasis efficiency [74,75,76]. However, it is important to notice that cancer dissemination, classically thought as a late process in cancer development, starts very early. In fact, over time, even small tumours can shed millions of circulating tumour cells (CTCs), which are now being exploited as a diagnostic tool (liquid biopsies). CTCs may then engraft in distant sites, including the bone marrow, becoming disseminated tumour cells (DTCs) [74,77]. Every step of metastatisation requires a specific set of molecules, some of which may be common among different solid cancers, such as matrix metalloproteinase (MMP)-9 in tumour intravasation [78,79,80,81,82,83], and some others being specific for different cancers or type of metastasis, such as PTHrP for skeletal metastases [73,84].

### 3.3. Premetastatisation

An important step in bone metastatisation is the establishment of a “premetastatic niche”, i.e., tumour cells preparing the bone microenvironment to host them, before “switching homes” and moving to bone [77]. Much research has been devoted to this topic in lung and liver metastasis, but research on the bone premetastatic niche (PMN) seems to be lagging behind, with only scarce reports present. A factor that seems to be important in the establishment of the bone PMN is lysyl oxidase (LOX), an enzyme that deals with crosslinking collagen and elastin in the extracellular matrix to increase tensile strength [85]. LOX has been shown to increase matrix stiffness, modulate the microenvironment and promote the establishment of a metastasis-permissive network of crosslinked fibres in bone, both in colon [86] and, importantly, in breast [87] cancer. On the other hand, before the concept of PMN was even coined, Kelly and colleagues found that primary tumour-derived heparanase was able to influence the bone microenvironment and increase bone resorption [88], which could prime the bone microenvironment for metastasis starting the vicious cycle from a distance before metastasis are established. Similarly, PTHrP, which normally acts in a paracrine fashion, can act from the primary tumour to cause a net increase in bone resorption, priming the bone microenvironment to metastasis, while causing tumour-induced hypercalcaemia [73,89]. TGF-β has also been shown to have a similar effect [90].

Bone marrow-derived cells seem to have a crucial role in establishing premetastatic niches in other organs [91,92,93,94]. Whether this is true for the bone marrow is still not demonstrated, although other niches located there are important for tumour dormancy and homing. Osteopontin (OPN), a bone matrix protein produced by osteoblasts, has also been studied in the field of bone metastasis for years [95]. In fact, OPN can also be expressed by cancer cells (because of osteomimicry [7]) and is a known driver of bone metastases, whose inhibition has even been hypothesised as a BM-inhibiting therapy [96]. It partakes in different stages of BM, including the establishment of the premetastatic niche, by increasing the expression of growth and chemotactic factors in the BM, such as b-FGF, hepatocyte growth factor (HGF) and TGF-β [95,96].

### 3.4. Homing

This process expresses the ability of cancer cells to set roots in a distant organ’s microenvironment, surviving in it, and either becoming dormant or proliferating. This process is obviously very complex, but many molecular mediators have been identified and characterised. A key player is αvβ3, an integrin that is well known for mediating the adhesion of osteoclasts to the bone matrix [26], which has been shown to favour bone homing and metastasis when expressed in breast cancer cells [97,98,99,100,101]. Similar actions have been proposed for very late antigen (VLA)-4, which interacts with vascular cell adhesion molecule (VCAM)-1, constitutively expressed in bone marrow endothelial and stromal cells [102]. Another possible player in cancer homing to bone is connective tissue growth factor (CTGF), which has been implicated in BM development in a seminal large-scale study more than 15 years ago [103]. This study exploited differently osteotropic cancer cell lines to identify factors that would be important for bone metastatisation by analysis of differentially expressed genes, and CTGF was one of the top hits. CTGF has been studied by different groups over the years with mixed results [104,105], and is now making a comeback in the context of prostate cancer, where it seems to drive a RUNX2-RANKL axis to promote BM and tumour progression [106].

An important topic, which still has momentum despite many years of research have already been put in it, is the commonality between the homing mechanism of haematopoietic stem cells (HSCs) and cancer cells [107]. A key molecular player seems to be C-X-C chemokine receptor type (CXCR)-4, the ligand for stromal-derived factor (SDF)-1. CXCR4 is crucial for haematopoietic stem cells (HSC) homing [108,109], but also cancer cells can exploit this pathway in order to exit the bloodstream and interact with the bone marrow stromal components [77,110,111].

CD44 is the major cell ligand for hyaluronan, and has been proposed as a stemness marker in breast cancer [112,113,114,115]. Intriguingly, both CD44 and αvβ3 are receptors for OPN, a major component of the bone matrix [115]. Therefore, CD44 may act in concert with αvβ3 integrin to home cancer cells to bone. Furthermore, CD44 can interact with MMP-9, providing the cancer cell with enhanced invasion potential [116]. Another function of this protein, which may be important for driving cancer cells in the bone marrow niche, is that CD44 can also bind CXCR4, which is crucial in retaining HSC in the niche, and might be important for cancer cells as well [117]. Annexin (ANXA) II can also bind SDF-1 [118] and is another crucial factor that helps localise HSC in the niche [119]. Unsurprisingly, this protein can be also exploited by cancer cells to “steal” the HSCs’ spot in the bone microenvironment [120,121]. Other factors, which have been linked to HSC and tumour dormancy will be presented in a separate section.

The involvement of the Notch pathway in bone metastasis has been poorly investigated, and only in recent years have specific reports emerged on the topic [122,123,124]. Jagged-1, a ligand for Notch family members, is expressed by metastatic cancer cells, and can bind Notch on osteoblasts and preosteoclasts. This, in turn, triggers the cleavage of the Notch intracellular domain (NICD) and its nuclear translocation, which activates downstream effectors of the pathway, eventually leading to IL-6 production by osteoblasts, and preosteoclast differentiation into mature osteoclasts, both directly and through IL-6 [123,124]. Despite a few years of relative quietness on the topic, Jagged-1 has proven itself to be a viable target in bone metastasis by Zheng and colleagues [122] especially in combination with chemotherapy.

Dickkopf Wnt signalling pathway inhibitor (DKK)1 is another novel factor in bone metastasis, which shows an interesting dichotomy in lung vs. bone metastasis. In fact, a recent report by Zhuang and colleagues [125] shows that DKK1-high tumours preferentially metastatise to bone, while DKK1-low tumours tend to migrate to the lungs. The dichotomy is maintained in the pathways hindered by DKK1: in lung this protein impairs noncanonical Wnt pathway, which would lead to immune cells chemotaxis and thus slower growth, while in bone it acts on osteoblasts to reduce canonical Wnt pathway activation [125], which tilts the osteoblast-osteoclast balance towards the osteoclasts, leading to increased bone resorption [125].

A relationship between PTHrP and CCL-2 (a.k.a. Monocyte Chemoattractant Protein-1, MCP-1) has emerged in the last decade and could be important in BM [126,127]. In fact, this protein is a potent osteoclastogenesis inducer, that can even overcome the antiosteoclastogenesis effect given by granulocyte-monocyte (GM)-CSF [128]. CCL-2 appears to be a “textbook” example of a vicious cycle protein. In fact, it is induced by PTHrP, induces osteoclastogenesis, and at the same time acts autocrinally to increase tumour production of vascular endothelial growth factor (VEGF)-A, eventually promoting angiogenesis and tumour growth [127,128]. However, inhibition of CCL-2 gave inconclusive results in reducing skeletal metastases, which shows just how complex and diverse the tumoural bone microenvironment is in patients [129,130].

### 3.5. Hypoxia, Angiogenesis and Blood Vessels in Bone Metastasis

Seminal works by Albert Lasker Basic Medical Research awardees and Nobel laureates Gregg L. Semenza, William G. Kaelin Jr and Sir Peter J. Ratcliffe [131,132] showed how important oxygen levels adaptation is in cells, which is mainly performed through the hypoxia-inducible factors (HIF) family [133]. This is true in physiological settings as well as pathological ones, such as cancer [134]. If we consider that arterial blood hold 130–80 mmHg and organs like kidney have 72 mmHg, we could define the endosteum as a hypoxic microenvironment, with pO_2_ dropping steeply to 50mmHg in close proximity to the endosteal niche [2,135]. Hypoxia seems to favour bone cells activity both directly, and through guaranteeing the coupling between angiogenesis and osteogenesis [136,137,138]. Unsurprisingly, the HIF family of factors and their many downstream targets are also important in breast cancer and BM. Recent evidence shows that HIF-1α signalling in osteoblasts increases breast cancer metastasis towards all districts, including bone [139]. The expression of the premetastatic factor LOX, discussed above, is strongly induced by HIF-1α [140,141], although it has been also reported to promote BM independently of HIF-1α in colon cancer [86]. In non-small-cell lung cancer (NSCLC), a correlation between HIF-1α and BM has been proposed as well in a retrospective study by Pezzuto et al. [142]. The intriguing aspect of this work is that HIF-1α positivity has been observed in the primary site rather than in the metastatic foci, characterising this factor not only as a local metastasis determinant but also as a player in the metastatic organotropism from the tumour cells’ side. Consistently, another previous study came to similar conclusions in breast cancer, showing that primary tumours that induce bone micrometastases have specific molecular signatures, which include the rat sarcoma (RAS) family and HIF-1α [143]. Moreover, experimental BM studies from almost 15 years ago reported that overexpressing a dominant-negative version of HIF-1α decreases BM development, whereas a constitutively active HIF-1α increases it, and targeting hypoxic cells with a caspase-3 activating protein selectively reduces BM [144,145].

In line with the key role of oxygen homeostasis in this context, we found that one of the most upregulated genes in bone metastatic samples from patients carrying also metastases to visceral organs vs. bone metastasis only patients was haemoglobin beta (*HBB*) [146]. Of course, this haem-protein is well known for its oxygen-binding and transport function in red blood cells, but its role in cancer was unknown. Further investigations revealed that *HBB* was able to increase cancer aggressiveness through HIF-1α, that it was increased in primary tumours of higher grade, and that blocking its oxygen-binding ability could abrogate some of the phenotypical effects observed [147]. We hypothesised this was due to what we called “oxygen-positive hypoxia”, a phenomenon through which oxygen is sequestered and rendered unavailable for the cell sensors, leading to the activation of HIF-1α target genes, such as VEGF [147]. Another excellent report by an independent group came to similar conclusions, although HIF-1α was not identified as the determinant of the phenotype. Nevertheless, the group found that oxidative stress was reduced in HBB-expressing cells, which is consistent with lower free intracellular pO_2_, and also agrees with our findings [146,148]. Arguably, the most evident consequence of bone metastasis is pain, and hypoxia has a role to play in this as well. In fact, HIF-1α activates the Warburg effect in cancer cells, which induces the release of lactate and protons in the bone microenvironment, which, in turn, signal to nociceptive terminals in the bone, causing pain [2,149]. Of course, another major source of protons is the osteoclast, which is activated by the vicious cycle, and targeting the V-ATPase reduces cancer-induced bone pain [149,150,151], also considering that tumour cells exploit V-ATPase to release protons in the bone microenvironment [152].

Another important aspect related to hypoxia and cancer is sustained angiogenesis. This is one of the hallmarks of cancer, and the concept that hypoxia increases angiogenesis has been known for more than 15 years [153]. In fact, HIF-1α can activate the expression of VEGF and its receptors, along with Angiopoietins, bFGF, PDGF, stem cell factor (SCF), osteopontin (OPN), MMPs, nitric oxide synthase (NOS)-2, cyclooxygenase (COX)-2 [154] and ET-1 (which is also important for osteoblastic bone metastases [67,69]) [155,156]. However, apart from the obvious advantage of having a vasculature transporting nutrients and oxygen, the bone metastatic tumour has other less obvious advantages that derive from activating the angiogenic program in the bone marrow: the establishment of the vascular niche [157], which will be discussed in the dormancy section and the ability of many angiogenic factors to fuel the vicious cycle. Key examples are VEGF, which on one hand promotes osteoblast differentiation and activity and can, therefore, fuel the osteoblastic vicious cycle [158,159,160,161] while, on the other hand, it activates osteoclastogenesis, potentially fuelling the osteolytic vicious cycle as well [162]. Similarly, bFGF can trigger osteoclast differentiation but also osteoblast activity, and, therefore, may contribute to both vicious cycles in BM [163,164,165,166].

### 3.6. Tumour Cellular Dormancy

Tumour dormancy is a behaviour that some cancer cells adopt, by which they remain dormant as single cells or small clones. This is achieved by either stopping to proliferate, or by equalising the rate of cell death with that of proliferation [167]. This status is akin to what fungi or bacteria do when they become spores, since dormant cancer cells are highly resistant to many external insults: they develop multidrug and radiotherapy resistance, are able to elude classical chemotherapy because they are not proliferating as well as the immune system, and they can be reactivated when a suitable stimulus/i (which are only partially known) is applied [167,168]. Perhaps the most incredible aspect of this process is that the cell can stay dormant for many years, usually between 5 and 20, as disseminated tumour cell (DTC) [169] before cancer tragically relapses and is often extremely hard to treat, because it arises from such a highly resistant clone [167,168,170,171].

The bone marrow is likely the most common site where cellular dormancy occurs, since many of the mechanisms that are thought to keep cancer cells dormant, are based on molecular pathways that are usually used by haematopoietic stem cells for maintaining their own quiescence [167,168,170,172]. Undoubtedly, cellular dormancy is an extremely complex phenomenon, that reflects the complexity of the bone marrow microenvironment and the many niches that reside there. These are often just a few microns away from one another, so one cell can participate in more than one niche at the same time. There are at least two niches in the bone marrow that allow cancer cells to be kept quiescent, and that rely on cell-cell contact as well as soluble factors: the endosteal niche and the vascular niche. Since the two are likely to coexist and cooperate in the bone marrow microenvironment, they will be discussed as one for the purpose of this review. This niche is formed by tumour cells, HSCs, MSC-osteoblast lineage cells, osteoclasts, endothelial cells and other players such as osteocytes and bone marrow adipocytes, which can all influence tumour cells dormancy [57].

In the early years, much work was done on prostate cancer cell dormancy. One of the first factors that was identified in cellular dormancy was BMP-7, which induces a reversible proliferation arrest in bone metastatic prostate cancer cells, through p21, p38 and n-myc downstream-regulated gene (NRDG)-1 [173]. This growth arrest could be reverted by BMP-7 removal, thus resulting in overt metastasis. Intriguingly, several years later an independent report showed that osteonectin activation in MSC-lineage cells promotes BMP-7, which could then lead to prostate cancer dormancy [174].

Another factor that proved to be important in tumour dormancy is tank-binding kinase 1 (TBK1). This is a noncanonical IKK family member that mediates part of the toll-like receptor-mediated innate immunity, leading to the transcription of target genes such as RANTES and interferon-β [175]. Intriguingly, Kim and colleagues found that prostate cancer cells contacted osteoblasts in the bone marrow, they activated TBK-1, eventually leading to an inhibition of the target of rapamycin (mTOR) pathway. This, in turn, gives rise to an arrest of protein synthesis and growth, drug resistance, and increased “stemness”, which fits the definition of cellular dormancy to the letter [175]. In breast cancer, other factors have been identified as important in determining niche-mediated cellular dormancy, such as N-cadherin. It is expressed in cancer cells after epithelial to mesenchymal transition, but also by a subset of osteoblasts called spindle-shaped N-Cadherin-positive osteoblasts (SNOs) [176,177,178]. Intriguingly, SNO cells have been implicated in the maintenance of HSC quiescence [179], albeit the role of N-cadherin (CDH2) has been ruled out as a determining factor [180] per se, while ablating all CDH2-positive cells using diphtheria toxin receptor models, caused a tremendous mobilisation of HSC, confirming that SNO cells are indeed important in the maintenance of the HSC bone marrow niche [179]. We found that SNO cells were able to reduce the proliferation of breast cancer cells, especially when selected for Notch2, consistent with the fact that SNO cells expressed high levels of the Notch ligand Jagged1 [176]. However, Abravanel and colleagues obtained different results when investigating the Notch family [181], likely because fine-tuning such an important pathway might cause dramatic differences, as is shown in other reports in the literature [176]. Another pathway that is still under investigation in this regard is the already cited SDF-1/CXCR4 pathway, which might partake in this process as well, although there is currently no consensus on the matter, and results seem to be pointing at different directions in different cancers [182,183,184,185].

Besides the knowledge of the molecular mechanisms allowing tumour dormancy, a crucial and very important issue is to identify which stimuli can revoke it, awakening the cancer cell into an overt metastasis. A general consideration is that the extracellular signal-regulated kinase (ERK) pathway has a crucial role in determining whether a cancer cell will remain dormant (inactive ERK pathway) or proliferate (activated ERK pathway). ERK inactivation is also linked to endoplasmic reticulum (ER)-stress, which also makes dormant cancer cells chemotherapy-resistant [186,187,188]. A number of studies suggest that integrin signalling might be crucial for the switch from a dormancy state to a reactivated metastasis. In particular, loss of integrin β1 signalling has been reported to be a key factor not only in the proliferation of primary and disseminated tumours [189,190,191] but also in the reactivation of dormant cancer cells, acting also through the Focal adhesion kinase (FAK)/SRC/ERK pathway [192]. Therefore, β1-integrin could be a potential therapeutic target, which can be exploited to keep tumour cells dormant, using neutralising molecules such as ATN-161, volociximab and JSM6427 [193]. Discoidin domain receptor (DDR)-1 can also trigger reactivation of cancer at multiple secondary sites including lung, brain and bone. This happens through tetraspanin transmembrane 4 L six family member 1 (TM4SF1), acting through the PKCα/JAK/STAT pathway to promote cell proliferation [194]. Intriguingly, DDR-1 noncanonical signalling may also be activated by binding to collagen, which could be another possible means of cancer cell awakening in bone. In fact, bone is highly enriched in collagen, which is usually buried in the mineral matrix, but can be exposed following osteoclastic bone resorption or acidification of the microenvironment [194]. Wnt signalling promotes G1-S transition through Cyclin E1 and D1, and c-myc [195] and Dikkopf-1 (DKK1), a Wnt signalling inhibitor, has been reported to promote tumour dormancy through an autocrine [196], but probably also paracrine signalling network. Factors that can have an opposite effect on the Wnt pathway, such as tenascin C and periostin (albeit indirectly) have opposite effects on tumour dormancy and can promote metastatic reactivation [197]. Finally, also inflammation can trigger reactivation of dormant cancer cells in distant sites, consistent with the fact that high circulating levels of acute-phase proteins correlate with distant-site recurrence in breast cancer [198].

### 3.7. Extracellular Vesicles in Bone Metastasis

Extracellular vesicles (EVs) are particles ranging from 20 to 1000 nm of diameter, which are delimited by a phospholipid bilayer that is compositionally and structurally reminiscent of a plasma membrane [199]. EVs contain multimolecular cargoes that can mirror the composition of the origin cell, or be enriched of specific molecules, such as miRNAs and other noncoding RNA, mRNAs, DNA, and specific membrane proteins, such as CD63, the most highly used marker of EVs. They have been implicated in a variety of physiopathological events and can be used by virtually every cell type as means of paracrine as well as long-distance communication [200,201]. Unsurprisingly, cancer cells can use EVs to influence their microenvironment, promoting tumour progression, metastasis, and organotropism [202]. A seminal report by Peinado et al. [203] showed that exosomes from melanoma cells could reprogram bone marrow progenitor cells, which would then migrate out and into the metastatic site, establishing a premetastatic niche in the target organ.

A large body of evidence is accumulating that exosomes can influence metastatic behaviour of many types of cancer, in different secondary sites [202]. We recently showed that EVs are indeed able to influence bone biology, and osteoblast-EV-shuttled RANKL is a novel way through which osteoblasts promote osteoclast survival without direct cell contact [204]. We also found that osteotropic breast cancer cells (MDA-MB-231) can reprogram osteoblasts so that they produce a higher amount of RANKL-EVs, which contributes to the vicious cycle by inducing osteoclastogenesis. Furthermore, we showed that MDA-EVs are able to also increase osteoclastogenesis directly, and that they strongly inhibit osteoblast activity, while activating angiogenesis in the bone marrow microenvironment [205]. This means that through a single delivery system, osteotropic cancer cells may be able to radically reprogram the bone microenvironment to their advantage, thereby increasing metastatic growth. Intriguingly, we show that systemic administration of MDA-EVs in mice results in the targeting of bone cells (osteoblasts and osteoclasts) with high specificity vs. the bone marrow, which only shows a small percentage of positive cells [205].

Another strong indication of the importance of EVs in metastatic tropism comes from Hoshino and colleagues, who showed in a very important paper for the field, that integrins present on the membrane of EVs are able to tell the organotropism of the origin tumour when it metastasises, because according to the integrin set, EVs will reprogram a specific microenvironment turning it into a permissive premetastatic niche [206]. In fact, the authors show that preconditioning mice with EVs presenting integrins associated with lung metastasis, resulted primarily in lung metastasis upon intracardiac injection of MDA cells, even if these were of a highly selected bone-targeting clone [206]. Nevertheless, the authors could not correlate any specific EV-integrin to BM. Other indications come from recent reports showing that lung cancer-derived EV-mediated transfer of Amphiregulin [207] or miR-21 [208] promote osteoclastogenesis, while another report identified an EV-miR signature enriched in metastatic lung cancer [209]. In prostate cancer, some advances in this field were also achieved, with Karlsson and colleagues finding that TRAMP-C1-EVs impaired osteoclastogenesis [210] and Inder et al. showing that Cavin-1/PTRF can alter the EV content of PC, which modifies its osteoblast- and osteoclast-activity-influencing behaviour [211]. Intriguingly, MSC-EVs have been shown to promote a dormancy-like proliferation reduction in metastatic breast cancer cells via miR-23b in vitro, which speaks volumes about the versatility of these multimolecular messengers, that could have a crucial role in bone metastasis.

### 3.8. Role of Immune Cells in Bone Biology and Metastases

Immune cells are among the most important regulators of bone physiology and pathology, as testified by the immense body of literature in the field of “osteoimmunology”, a term referred to the cross-regulation of bone and immune cells [212]. T-lymphocytes, especially Th17 cells, are strong inducers of osteoclastogenesis, both directly and indirectly through osteoblast induction of RANKL and M-CSF [213,214], while the immunosuppressive T-reg cells are antiosteoclastogenic [25]. B-cells produce RANKL, which acts as both an autocrine factor and an osteoclastogenesis inducer, and in fact, B-cell deficient mice are protected against ovariectomy-induced bone loss. Additionally, neutrophils can influence bone physiology, mainly by cytokine secretion and by attracting Th17 lymphocytes in situ [215]. Unsurprisingly, all these immune cells present in the bone marrow, influence the fate of bone metastatic cells in addition to influencing bone biology.

Circulating osteoclast precursors increase in number in bone metastatic patients, and coculturing them with T-cells results in osteoclastogenesis with no further stimulation by RANKL or M-CSF [216]. Furthermore, IL-7, a key cytokine produced by immune cells in the tumour context, is able to increase RANKL and other inflammatory cytokines such as TNFα in osteoblasts and bone marrow stromal cells, thus increasing osteoclastogenesis and fuelling bone degradation in the metastatic context [217,218,219]. Importantly, the RANKL-RANK contact between T-cells and tumour cells promotes tumour growth, invasion and metastasis [220]. To consolidate the link between T-cells and tumour burden in bone, increasing T-cells response by knocking out Lyn tyrosine kinase, causes a reduction in bone metastatic growth, despite increased osteoclastic bone resorption [221,222]. Another important cell type in metastatic bone growth is the myeloid-derived suppressor cell (MDSCs). This is a myeloid cell type characterised by coexpression of Gr-1 (od CD33 in humans) and CD11b, which can suppress both innate and adaptive immunity, thus increasing tumour growth in multiple cancers, including lung, breast and multiple myeloma [223,224]. It is, therefore, unsurprising that cells are a negative prognostic factor in these cancers, also considering they are precursors for osteoclasts and can differentiate into OCS after stimulation [225]. MDSCs are also able to activate tumour angiogenesis by secretion of VEGF and bFGF, which can be directly incorporated in the tumoural endothelium [226,227]. Immune cells are also involved in the establishment of two different types of tumour dormancy. On the one hand, potential dormant tumour cells have been reported to compete with haematopoietic stem cells (HSCs) for the endosteal niche [176,228], where they become dormant (see Section 3.6). On the other hand, immune cells are also able to entertain a fight with metastatic (or primary) cancer cells, balancing the rate of tumour proliferation with that of immune-mediated killing, thus inducing an apparently latent state in the tumour mass. This process is termed immune-mediated dormancy [229,230]. This equilibrium status seems to be mainly promoted by adaptive immune cells and factors, such as CD4^+^/CD8^+^ T-cells, perforin-mediated toxicity by NK cells, IL-12 and IFN-γ, while cancer cells respond by increasing Treg and MDSC production of TGF-β, IL-10, ROS and NO and upregulating their own programmed death-ligand 1 (PD-L1) expression [229,231]. Moreover, neutrophils and macrophages have been shown to be alternatively activated by cancer cells into an M2 or N2 phenotype, respectively, thus becoming tumour-associated neutrophils (TANs) or macrophages (TAMs), eventually leading to increased angiogenesis and metastatic dissemination in secondary sites including bone [232,233] Although interesting, this is surely not the full extent of cross-regulation between cancer cells and immune cells in the bone microenvironment. After all, if a tumour becomes able to grow in the immune “lion’s den” of the organism, its action on immunity cannot be anything short of incredible.

## 4. Discussion and Conclusions

**Figure 1:**
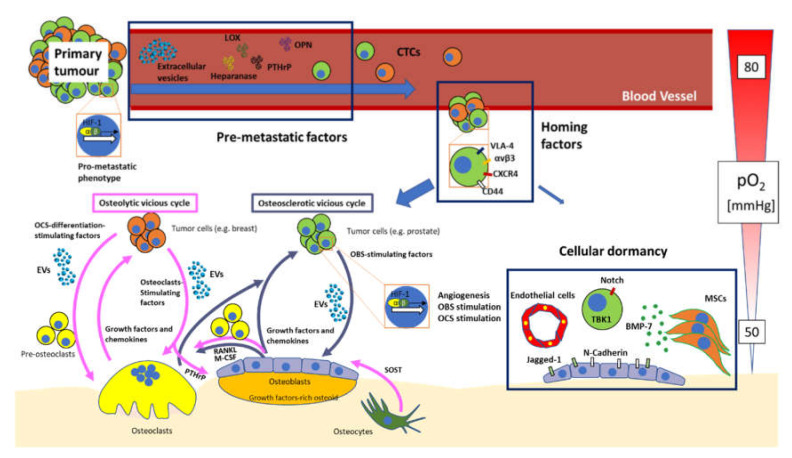
Overview of the bone metastatisation process. The primary tumour gets access to the circulation, also thanks to the activation of the HIF pathway, which activates a pro-metastatic program in a subset of cancer cells. Before metastases take place, a number of premetastatic factors, including extracellular vesicles, lysyl oxidase (LOX), heparanase, parathyroid hormone-related peptide (PTHrP), and osteopontin (OPN) educate the bone microenvironment making it suitable for metastatic engraftment. Circulating tumour cells (CTCs) can then extravasate and engraft in the bone thanks to homing factors such as very late antigen (VLA)-4 and αvβ3 integrins, C-X-C chemokine receptor type (CXCR)-4 and CD44. Once in the bone microenvironment, most cells entertain a cross-communication with bone cells, changing the physiologic “virtuous cycle” between osteoblasts and osteoclasts into a “vicious cycle” that favours tumour growth. Two types of vicious cycles are known. The osteosclerotic vicious cycle, normally established by prostate cancer cells, exploits osteoblasts (OBS)-stimulating factors (including extracellular vesicles) to increase OBS production of growth factors, as well as receptor activator of nuclear factor kappa B ligand (RANKL) and macrophage-colony-stimulating factor (M-CSF), that, in turn, stimulate osteoclast differentiation, further allowing the release of growth factors from the bone matrix. The growth factors produced by OBS and released by OCS then signal back to cancer cells, increasing their growth, thus closing the vicious cycle. The osteolytic vicious cycle is established by many types of tumours, including breast cancer. In this cycle, tumour cells use osteoclast-stimulating factors (including extracellular vesicles) and osteoclast differentiation-stimulating factors (including extracellular vesicles) to increase bone resorption, leading to the release of growth factors from the bone matrix, thus furthering tumour growth and restarting the vicious cycle. PTHrP is also exploited by breast cancer cells to induce osteoclastogenesis indirectly, through osteoblastic RANKL and M-CSF. Osteocytes may also take a part in the osteolytic vicious cycle, by suppressing OBS activity through secretion of sclerostin (SOST). A crucial factor in the vicious cycle is HIF, that is activated partially thanks to the low pO2 of the bone marrow microenvironment, and causing increased angiogenesis, OBS stimulation and OCS stimulation. A very small contingent of cells may undertake an alternative path and progress through cellular dormancy. This process requires the stimulation of many membrane and soluble factors, such as bone morphogenic protein (BMP)-7, that induces prostate cancer dormancy, or Jagged-1 which proposedly promotes dormancy through Notch-2 in breast cancer cells. Mesenchymal stromal cells (MSCs), endothelial cells and spindle-shaped N-cadherin-positive osteoblasts (SNOs) seem to be crucial in this process, but also other cell types may be taking part in this process too.

Here, we presented an overview of key topics related to bone metastasis (summarised in Figure 1), focusing our attention on classical concepts related to bone marrow metastasis, as well as aspects that have emerged in the very last few years, such as the cellular dormancy and extracellular vesicles. Factors of key importance regulating the establishment of the bone marrow premetastatic niche have been presented. An additional phenomenon that we discussed in this review, is cancer cells homing and establishment of the vicious cycle in the bone marrow. This is one of the most investigated areas in bone metastasis, with many seminal reports showing the importance of factors such as αvβ3 and VLA-4 integrins, OPN, CD44, CXCR4 and more.

Hypoxia is another crucial factor in bone metastasis development. The bone marrow is a fundamentally hypoxic environment, and HIF-1α is able to promote bone metastatisation even when expressed in the primary site. HIF modulators such as HBB may also take part in bone and visceral metastatisation. The HIF family may also activate proton production in the bone microenvironment, thus signalling to nociceptive terminals and causing severe pain, which is one of the most terrible consequences of bone metastasis. Cancer cells also exploit HIF signalling to activate angiogenesis, which results in the production of factors like VEGF and bFGF, which can not only activate angiogenesis, but also osteoblast and osteoclast activity, which can fuel the osteoblastic and osteolytic vicious cycles.

With regards to cancer cellular dormancy and extracellular vesicles, these topics are very complex, and despite considerable efforts by the scientific community, there is still much work to do before we reach a reasonable comprehension of these two aspects, which are clearly important in the cancer field. In particular, understanding how cellular dormancy works may allow us to create drugs that keep the cells dormant, or awaken them so that they become sensitive to chemotherapy. As for extracellular vesicles, they have potential both as biomarkers and therapeutic drug delivery systems. The fact that EVs mirror the content of the cell of origin, has been exploited in cancer theranostics to detect the onset of target therapy resistance or efficacy by circulating cell-free DNA analysis [234,235,236,237] in order to adjust therapy in itinere and in an individualised manner. Cancer cells produce very high amounts of EVs compared to their normal counterparts [199] and are enriched in aggressiveness-related features in patients. This also holds true for high invasive MDA-MB-231 vs. low invasive MCF7 cell lines [238,239]. Although in general terms, inter-patient variability seems to be in line with that of other biomarkers [240], a biomarker-based pilot study to assess normal levels of EV-shuttled factors is crucial for most biomarkers (e.g., miRNAs), since the field is new, and normality ranges, biological variability and technical variability are still not established [241,242]. However, the fact that these are communication devices for cells, opens many possibilities in the drug delivery field. In fact, loading extracellular vesicles with drugs, could allow us to minimise side effects, while increasing the drug concentration in target cells. This would be particularly important in bone metastasis, since the endosteal niche is hypoxic and relatively less perfused, and targeting would help increase therapeutic efficacy of cytotoxic drugs as well as targeted therapy.

Much work has been carried out on the topic of bone metastases, with the identification of useful therapeutic options, such as bisphosphonates, and Denosumab (anti-RANKL mAb), both targeting the osteoclastic side of the vicious cycles, and being able to reduce skeletal-related events both in metastatic prostate [243] and breast [244] cancers. Bone metastases are, therefore, treatable, but still incurable. Nevertheless, ongoing research holds much promise for the future, and will try to provide novel actionable pathways and drugs, in hopes of overcoming this very common complication of the most prevalent human cancers.

## Abbreviations

ANXA	Annexin
BALP	Bone-specific alkaline phosphatase
BM	Bone metastasis
BMA	Bone marrow adipocyte
BMM	Bone Marrow Macrophages
BMP	Bone morphogenetic proteins
BSP	Bone sialoprotein
CaSr	Calcium-sensing receptor
CCL	CC chemokine ligands
CCR	CC chemokine receptor
CDH2	N-cadherin
COX	Cyclooxygenase
CTCs	Circulating tumour cells
CTGF	Connective tissue growth factor
CXCR	C-X-C chemokine receptor type
DDR	Discoidin domain receptor
DKK1	Dikkopf-1
DTCs	Disseminated tumour cells
ER	Endoplasmic reticulum
ERK	Extracellular signal-regulated kinase
ET	Endothelin
EVs	Extracellular vesicles
FABP4	Fatty acid-binding protein 4
FAK	Focal adhesion kinase
FGF	Fibroblast growth factors
HBB	Haemoglobin beta
HGF	Hepatocyte growth factor
HIF	Hypoxia-inducible factors
HSC	Haematopoietic stem cells
IGF	Insulin-like growth factor
IL	Interleukin
LOX	Lysyl oxidase
MCP	Monocyte Chemoattractant Protein
M-CSF	Macrophage-colony stimulating factor
MDSC	Myeloid-derived suppressor cells
MITF	Microphthalmia Transcription Factor
MMP	Matrix metalloprotease
MSCs	Mesenchymal stromal cells
NFATc1	Nuclear factor of activated T-cells, cytoplasmic, calcineurin-dependent
NfĸB	Nuclear factor kappa B
NICD	Notch intracellular domain
NO	Nitric Oxide
NOS	Nitric oxide synthase
NRDG	N-myc downstream-regulated gene
NSCLC	Nonsmall-cell lung cancer
OCN	Osteocalcin
OPN	Osteopontin
OPN	Osteopontin
PDGF	Platelet-derived growth factor
PD-L1	Programmed death-ligand 1
PMN	Premetastatic niche
PTHrP	Parathyroid hormone-related peptide
RANKL	Receptor activator of nuclear factor kappa B ligand
RAS	Rat sarcoma
Runx2	Runt-related transcription factor 2
SCF	Stem cell factor
SDF	Stromal-derived factor
SNOs	Spindle-shaped N-Cadherin-positive osteoblasts
SOST	Sclerostin
SREs	Skeletal-related events
TAM	Tumour-associated macrophages
TAN	Tumour-associated neutrophils
TBK1	Tank-binding kinase 1
TGF-β	Transforming growth factor-beta
TM4SF1	Tetraspanin transmembrane 4 L six family member 1
TNF-α	Tumour necrosis factor-alpha
TOR	Target of rapamycin
VCAM	Vascular cell adhesion molecule
VEGF	Vascular endothelial growth factor
VLA	Very late antigen
Wnt	Wingless-type MMTV integration
